# Evaluation of EEG Features in Decoding Individual Finger Movements from One Hand

**DOI:** 10.1155/2013/243257

**Published:** 2013-04-24

**Authors:** Ran Xiao, Lei Ding

**Affiliations:** ^1^School of Electrical and Computer Engineering, University of Oklahoma, Norman, OK 73019, USA; ^2^Center for Biomedical Engineering, University of Oklahoma, Norman, OK 73019, USA

## Abstract

With the advancements in modern signal processing techniques, the field of brain-computer interface (BCI) is progressing fast towards noninvasiveness. One challenge still impeding these developments is the limited number of features, especially movement-related features, available to generate control signals for noninvasive BCIs. A few recent studies investigated several movement-related features, such as spectral features in electrocorticography (ECoG) data obtained through a spectral principal component analysis (PCA) and direct use of EEG temporal data, and demonstrated the decoding of individual fingers. The present paper evaluated multiple movement-related features under the same task, that is, discriminating individual fingers from one hand using noninvasive EEG. The present results demonstrate the existence of a broadband feature in EEG to discriminate individual fingers, which has only been identified previously in ECoG. It further shows that multiple spectral features obtained from the spectral PCA yield an average decoding accuracy of 45.2%, which is significantly higher than the guess level (*P* < 0.05) and other features investigated (*P* < 0.05), including EEG spectral power changes in alpha and beta bands and EEG temporal data. The decoding of individual fingers using noninvasive EEG is promising to improve number of features for control, which can facilitate the development of noninvasive BCI applications with rich complexity.

## 1. Introduction

Brain-computer interface (BCI) is an assistive technology, which decodes neurophysiological signals from the human brain and translates them into commands to control external devices, such as computer programs, electrical wheelchairs, and neuroprosthesis [1–4]. For people with severe motor disabilities, BCI provides an alternative approach to communicate with the external world without going through damaged motor output pathways [5, 6].

In terms of measurements utilized, BCIs can be categorized into invasive and noninvasive ones. Invasive BCIs mainly use local field potential (LFP) and electrocorticography (ECoG) [7–12]. Both techniques record neuroelectrical activities of the brain with high spatiotemporal resolution and signal-to-noise ratio (SNR) [12], while the implantation of electrodes poses a potential risk for BCI users. On the other side, noninvasive BCIs take advantage of noninvasive measurements, for example, electroencephalography (EEG), magnetoencephalography (MEG), and functional magnetic resonance imaging (fMRI) [13–19]. All of them require no surgeries for applications. Among these noninvasive techniques, EEG has been widely adopted in BCIs [2–4, 13–15, 20], due to its merits of easy setup, mobility, and low cost, as well as providing signals with reasonable SNR and high temporal resolution.

Due to the advancements in biomedical equipment and signal processing techniques, the field of noninvasive BCI grows rapidly with several patterns of brain activities that have been identified and applied for noninvasive applications. The most popular ones include features extracted from motor execution/imagery of certain human body parts [13, 14, 21], event-related P300 [22, 23], steady-state visual evoked potentials (SSVEP) [24], and some others. Motor execution/imagery elicits power changes in alpha/beta bands, that is, event-related desynchronization/synchronization (ERD/ERS) [21], which have been widely used in cursor tasks and neuroprosthesis [3, 13, 25, 26]. However, a remaining challenge of applying movement-related features in noninvasive BCIs is the limited number of distinguishable patterns available in order to generate more control signals, which largely confines the complexity of noninvasive BCIs to only simple tasks.

During the past decade, many efforts have been made using EEG to decode movements of large body parts of the human. For example, Doud et al. decoded movements from upper limbs for continuous BCI control [27]; Gu et al. investigated the feasibility of discriminating type and speed of wrist movements [28]; Zhou et al. performed classification on movements from elbow and shoulder using EEG [29]; and one of our previous studies discriminated different types of motor imageries from both hands [30], only to name a few. To further increase the number of control signals for BCI, decoding movements of fine body parts, such as individual fingers from one hand, is a viable mean [31–34], while there are some difficulties, particularly with the use of noninvasive EEG. Compared to invasive measurements, the relatively poor spatial resolution of EEG makes it hard to decode individual finger movements, since they activate adjacent brain regions [35]. Furthermore, neural signals are further attenuated and smeared by the Dura mater, cerebrospinal fluid, and skull before reaching the head surface, which makes it even harder to discriminate movements from fine body parts using EEG. A recent study [36] has achieved promising decoding performance using temporal EEG data as features, when classifying movements from four fingers of one hand. It demonstrated the existence of discriminative information of individual finger movements in EEG. Another study uses information extracted from ECoG to decode individual finger movements [37], which are projections of spectral powers on spectral principal components (PCs) obtained by principal component analysis (PCA), and suggests a new type of decomposition for spectral feature extraction. While these studies provide possible approaches to extract features for decoding individual fingers from one hand, they are carried out under different experimental conditions and using different signals, making it hard to compare their performance in discriminating individual fingers. Furthermore, the efficacy of use of spectral PCs from EEG has not been demonstrated, particularly as compared with other features in decoding finger movements. The comparison of these features can provide a reference for feature extraction in such decoding tasks.

The present study evaluated three types of EEG features, including projections on spectral PC(s) (single PCs or multiple PCs), ERD/ERS (in both alpha and beta bands), and temporal data, when subjects performed the same task, that is, individual finger movements of one hand. Different features from a same set of channels were extracted and single-trial EEG data were then classified to decode individual fingers using a support vector machine (SVM) technique [38]. The decoding accuracies were statistically compared against the guess level and among different types of features. The confusion matrices classifying individual fingers using EEG were constructed. The present results indicated that EEG features using both spectral PC projection coefficients and temporal data produced decoding accuracies significantly higher than the guess level (*P* < 0.05), while the decoding accuracies using ERD/ERS features from individual frequency bands (i.e., alpha and beta bands) did not reach the significant level. The present results further suggested that the combined EEG features from the first three spectral PCs provide significant better decoding accuracies (an average accuracy of 45.2% across all subjects) for individual finger movements than all other features investigated (*P* < 0.05), which was supported by results in confusion matrices as well. These findings demonstrate a new way to extract EEG features for decoding individual fingers of one hand, which can facilitate the development of noninvasive BCI applications with rich complexity.

## 2. Materials and Methods

### 2.1. Subjects and Experimental Protocol

Six subjects (mean age: 27.3, range: 22~32, all right handed), who had no previous experience with the current experimental protocol, volunteered to participate in the study. All subjects provided written informed consents prior to taking up the experiments. The study was approved by the Institutional Review Board of the University of Oklahoma. 

**Figure 1:**
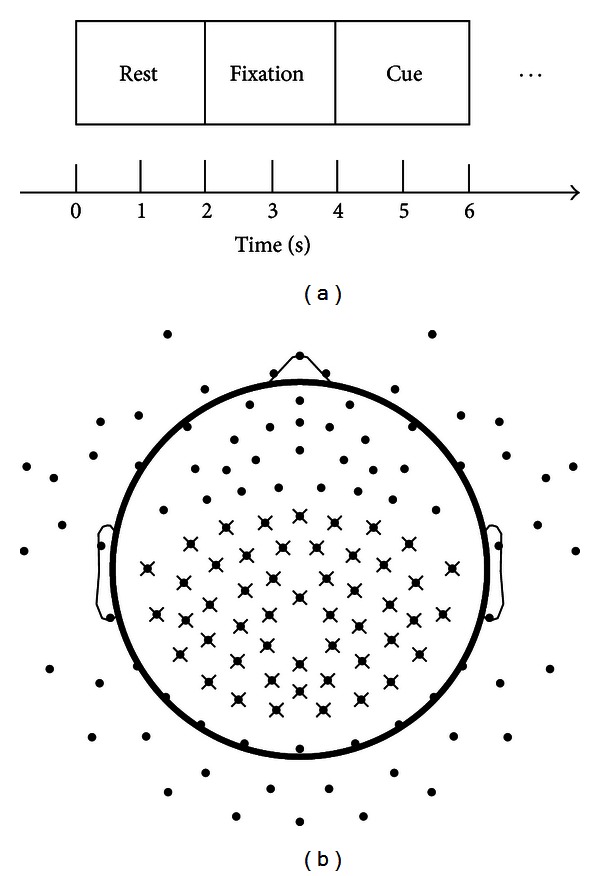
Experimental trial design and EEG sensor layout. (a) Each trial consists of three segments: 2 s for rest, 2 s for fixation, and 2 s for movement. (b) Channel locations for EEG sensor net. Each dot denotes an EEG sensor and each cross denotes a channel selected for decoding.

The experiments were conducted in a dim-lighted and electrically shielded chamber room to reduce environmental noise. During the experiments, subjects either rested or performed repetitive movements of individual fingers from one hand according to visually presented cues. The stimuli were presented using E-Prime software (Psychology Software Tools, Inc., Pittsburgh, PA, USA) as illustrated in Figure 1(a). In the first two seconds of the trial, the computer screen was black, allowing time of necessary blinking or swallowing for subjects. After that, a fixation appeared in the center of screen for two seconds. During this time window, subjects were instructed to sit still and stare at the fixation, which provided data for resting conditions without artifacts. In the last two seconds of the trial, one of five words (thumb, index, middle, ring, and little) was randomly chosen and presented on the screen, cueing subjects to perform repetitive movements of corresponding fingers. Most subjects finished one session including 80 trials for each finger in total 40 minutes. One subject reported difficulties in finishing the entire session and finished a session with 60 trials for each finger instead. Data from one subject were excluded from further analysis due to poor recording quality with large EEG artifacts. 

### 2.2. EEG Recording and Preprocessing

EEG data were acquired from a 128-channel sensor net using the Geodesic EEG System 300 (Electrical Geodesic Inc., OR, USA). The channel layout is depicted as black dots in Figure 1(b). The EEG signals were sampled at 250 Hz and referenced to a channel on vertex.

The raw EEG data recorded were firstly high-pass filtered at 0.3 Hz. A 60 Hz notch filter with 0.3 Hz transition band was then applied to the data to reduce the influence from power line noise. To further increase the SNR of data, an independent component analysis (ICA) was performed using the EEGLAB toolbox [39, 40] to remove independent components (ICs) related to electrooculogram (EOG), electrocardiogram (ECG), electromyogram (EMG), and other common artifacts. There were usually 10 to 20 ICs identified and rejected as artifacts in each subject. After temporal filtering, the EEG data went through a spatial filter named as common average reference (CAR), which could further increase the SNR of data [41]:

(1) $$\begin{array}{c} V_{\text{CAR}}\left(n,t\right)=V\left(n,t\right)-\frac{1}{N}\sum_{i=1}^{N}v\left(i,t\right), \end{array}$$

where *V*_CAR_(*n*, *t*) denotes the common average referenced potential at channel *n* and sample point *t*. It was calculated by subtracting EEG potential *V*(*n*, *t*) at channel *n* and sample point *t* to the average potential of total *N* channels at that sample point. 

After the previous preprocessing steps, the EEG data were segmented into 6-second epochs according to the trial structure depicted in Figure 1(a). One-second segment data centered at the last two seconds for movements, that is, 4.5–5.5 s of each epoch, were used to decode individual fingers, since maximal actions of fingers in most subjects were shown in this time window. 500 ms data from the onset of stimuli were not used for decoding, since subjects were preparing movements before execution [42]. In accordance with data for movements, their corresponding resting data were also selected as one-second segments, which were located in the middle of fixation, that is, 2.5–3.5 s of each epoch. Movement data and resting data, together with the corresponding labels that indicate the fingers moved, were then combined for later processing.

### 2.3. Feature Extraction

#### 2.3.1. Features from Spectral PCA Decomposition

The PCA method [43] was performed on EEG spectral powers to identify common spectral patterns across conditions. It transferred original signals into projections along uncorrelated principal components, which represented multiple common spectral patterns in all conditions. The use of PCA not only reduced the dimension of feature space but also identified features accounting for large variations in data. Both characteristics could improve decoding performance in classification problems.

The extraction of these spectral features involved multiple steps as the following. EEG segment data were firstly transferred into spectral powers, by calculating power spectral density (PSD) for each trial (both movement data and resting data) with

(2) Pn(f,m)=1T|∑t=−(T/2)(T/2)−1VCAR(n,t+T2+1,m)·H(t)          ·exp⁡(i2πT(f−1)t)|2,H(t)=(1+cos⁡⁡(2πt/T))2,

where *P*_*n*_(*f*, *m*) denoted PSD at channel *n* and frequency *f* for segment *m*. *H*(*t*) represented the Hanning window, with the window length *T* set as 250 sample points. The range of *f* was from 1 to 125 Hz, due to the sampling frequency of 250 Hz. 

Before performing PCA on data, the PSDs for each trial were normalized by

(3) $$\begin{array}{c} {\tilde{P}}_{n}\left(f,m\right)=\ln\left(P_{n}\left(f,m\right)\right)-\ln\left(\frac{1}{M}\cdot \sum_{p=1}^{M}P_{n}\left(f,m\right)\right), \end{array}$$

where the normalized PSD ${\tilde{P}}_{n}\left(f,m\right)$ was the log-transferred division between PSD of each segment data and the mean of all segment data (including movements and resting). The symbols *f*, *m*, *n*, and *M* denote frequency, segment, channel numbers, and the total number of segments, respectively. This operation compensated the uneven distribution characteristic of EEG spectral powers, which followed the power law and emphasized on low-frequency components. It also put the proportions of EEG spectral powers from 0 to 1 and from 1 to infinity on an equal footing, where one indicated the mean PSD of all segment data [37]. 

After the normalization, the spectral PCA decomposition started by calculating the second moment tensor of distribution function for EEG spectral powers [37] by

(4) $$\begin{array}{c} C\left(f,f^{\prime }\right)=\sum_{m=1}^{M}{\tilde{P}}_{n}\left(f,m\right)\cdot {\tilde{P}}_{n}\left(f^{\prime },m\right), \end{array}$$

where *f* and *f*′ were frequencies from 1 to 125 Hz. The constructed matrix measured how well spectral powers at two frequencies vary together along different segment data (i.e., different trials). Then, eigenvalues and eigenvectors of the matrix were calculated using a MATLAB (R2011a, the MathWorks, Natick, MA) function named “eig.” The eigenvectors were rearranged according to their corresponding eigenvalues in a descending order. These eigenvectors were spectral principal components (PCs) that represented different common spectral patterns in EEG spectral powers across conditions, ordered with decreasing significance. Each segment data was then projected onto these spectral PCs and the resulting projection coefficients were features used for classification.

The projection coefficients on the single PCs (i.e., the first PC and the second PC) and multiple PCs (i.e., combined first three PCs) were chosen to evaluate the decoding performance with features obtained from spectral PCs. The channels, marked by crosses in Figure 1(b), were selected for performing spectral PCA and decoding, which covered the posterior frontal cortex, motor cortex, and parietal cortex.

#### 2.3.2. Features from Individual Frequency Bands and Temporal Data

The extractions of both features from individual frequency bands and temporal data were performed on the same EEG segments used for spectral PCA analysis for the purpose of comparison. The first step in acquiring features in individual frequency bands was to transfer temporal EEG data into spectral powers in the frequency domain, which was achieved using (2) as well. After that, the spectral powers at 8–12 Hz (i.e., alpha band) and 13–30 Hz (i.e., beta band) were selected as movement-related features to decode individual finger movements. For using temporal data as movement-related features, EEG potentials from all movement segments were downsampled to 25 Hz by choosing one sample out of every ten samples, which was adopted from a previous study [36]. This operation reduced the computational workload for decoding tasks later, leaving 25 samples in each segment as decoding features.

### 2.4. Permutation and Classification

To evaluate decoding accuracies, the sequence of segments was randomly permuted 30 times before feature extraction and classification. The corresponding labels were permuted as well, in line with the segments. After each permutation, 80% of the data were selected as the training set and the remaining as the testing set. The features being evaluated in the present study were obtained only from the training set, which made sure that no data in the testing set were involved in building classifiers. 

A support vector machine (SVM) classifier [44] was adopted for decoding. The SVM classifier used a kernel method to map training data into a high dimensional space, where different classes of data could be linearly separated. Next, it searched for a hyperplane, which maximized the margin constructed by support vectors among different classes. The acquired hyperplane then served to distinguish data from different classes. In the present study, the LIBSVM toolbox was implemented using radial basis function (RBF) as kernel function [38]. For different features evaluated, the paired feature data and their labels in the training set were used to train the SVM classifiers. The trained classifiers were then used to predict labels of EEG segment data in the testing set. Finally, predicted labels were compared to true labels for these segments in the testing set to compute decoding accuracies with the use of different types of features.

### 2.5. Evaluation of Decoding Performance

To evaluate the performance of decoding individual finger movements using these features, the decoding accuracies acquired by different features were compared with the guess level using one-sample *t*-test. The guess level for 5-class classification problems was 20%. To compare the decoding performance of each pair of features, pairwise *t*-tests were performed on decoding accuracy data obtained from them. All *t*-tests were implemented using a MATLAB function named “*t*-test.” In addition, confusion matrices were constructed from data of decoding accuracies using each type of features to assess their decoding accuracies on individual fingers as well as their structures of misclassification.

## 3. Results

### 3.1. Features from Spectral PCA Decomposition

**Figure 2:**
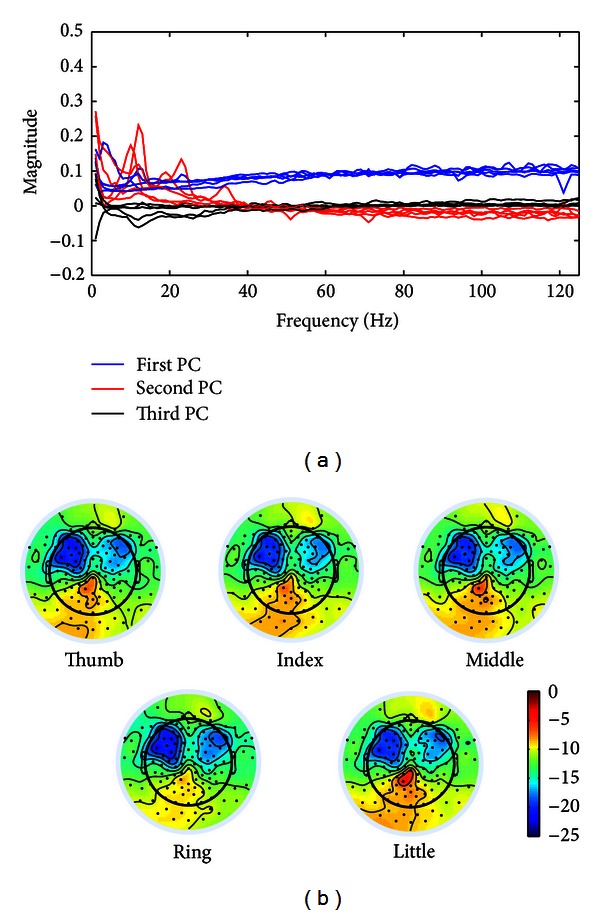
Spectral PC features from PCA decomposition. (a) Profiles of the first three PCs. (b) Topographies of projection coefficients on the first PC from movements of different fingers.

**Figure 3:**
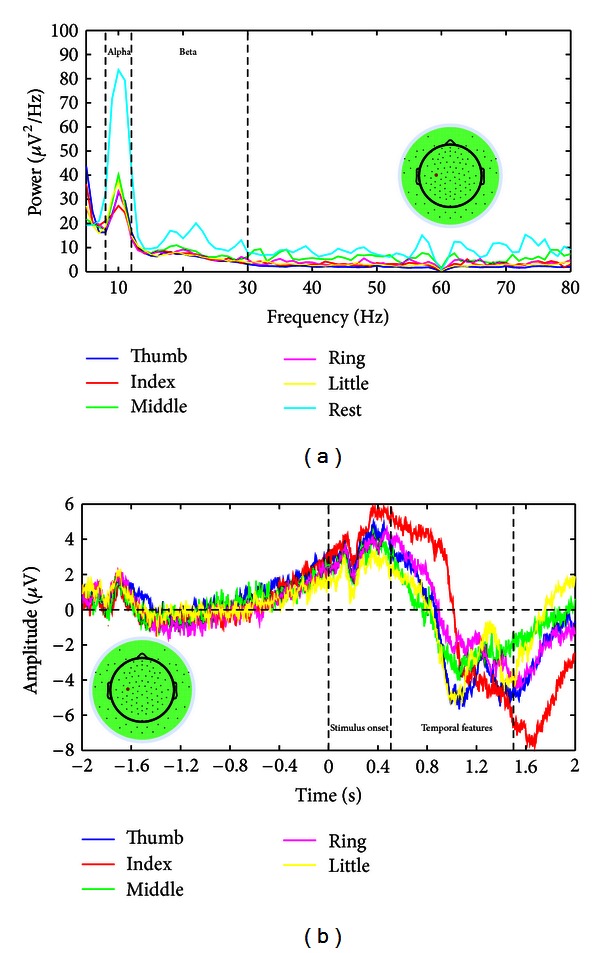
Illustration of averaged alpha/beta power changes and averaged temporal EEG data from a channel (the red dot) over the left motor cortex. (a) Average spectral powers as a function of frequency calculated from movement and resting segment data. (b) Average temporal EEG data from different fingers. Zero indicates the onset of movement cues.

Features from spectral PCA decomposition were evaluated from two aspects, that is, the profiles of PCs (i.e., the amplitude structure as a function of frequency) and the spatial patterns of projection coefficients over EEG electrodes (Figure 1(b)) for different fingers.  Figure 2(a) presents the profiles of the first three spectral PCs, which account for most variations in data (over 90% in average among all subjects). The first PC for each subject (blue curves) presents a broadband phenomenon, which is flat and positive across all frequencies. The phenomenon is consistent with results in the recent ECoG study [37], suggesting that the similar spectral pattern indicative of finger movements can be identified in EEG as in invasive ECoG. The second PC (red curves) mainly peaks at alpha band and beta band, which suggests the similar ERD/ERS phenomena within alpha and beta bands as discussed in Section 2.3.2 (also see Figure 3(a)). The third PC (black curves) exhibits small variations in low-frequency bands, which may represent some residual activities in low-frequency bands besides the first two PCs.

To evaluate the spatial patterns of features from the spectral PCA, projection coefficients on the first PC for different fingers were plotted in Figure 2(b). It reveals that the projections of EEG data from different brain regions on the same spectral structure (as denoted by the spectral PC) present distinct patterns during finger movements. Projection coefficients on electrodes over both left and right motor cortices have large negative values, while they are more towards zeros on electrodes over the parietal cortex. More importantly, these distinct patterns covering different brain regions elicit variations to certain extents when moving different fingers from the same hand, with more obvious phenomena on the parietal cortex.

### 3.2. Features Using Spectral Powers and Temporal Data

All subjects moved fingers from the right hand, which elicited power changes in channels from the motor cortex on the left motor cortex (i.e., the contralateral side) [6, 21]. Hence, spectral powers averaged over all segments belonging to one condition from a channel on the left motor cortex were chosen to display (Figure 3(a)). The selected channel was marked by the red dot on the scalp map. It shows that all finger movements elicit power decreases in both alpha band (enclosed by 1st and 2nd vertical lines) and beta band (enclosed by 2nd and 3rd vertical lines) compared to the resting (denoted by the cyan curve), while spectral powers in alpha band present much larger decrease. However, no major differences in spectral powers among different finger movement conditions can be readily identified in both frequency bands. This observation suggests features of spectral powers from individual frequency bands may not suffice the task of decoding movements of fine body parts, that is, individual finger movements.

The same channel on the left motor cortex was selected to present features in temporal data averaged over all segments belonging to one finger aligning to the onset of movements (Figure 3(b)). The first vertical line indicates the onset of movement cues, and the following two vertical lines define the segments of data selected for extracting temporal patterns to decode individual finger movements. Average temporal waveforms from movements of different fingers are depicted using different colors. It presents similar EEG patterns prior to onset of movements, while distinct fluctuations in amplitudes are shown from different finger movements after stimulus onsets, particularly in the window selected for decoding. This difference in temporal data is utilized to decode individual finger movements from one hand. 

### 3.3. Classification Accuracies Using Different Features

**Figure 4:**
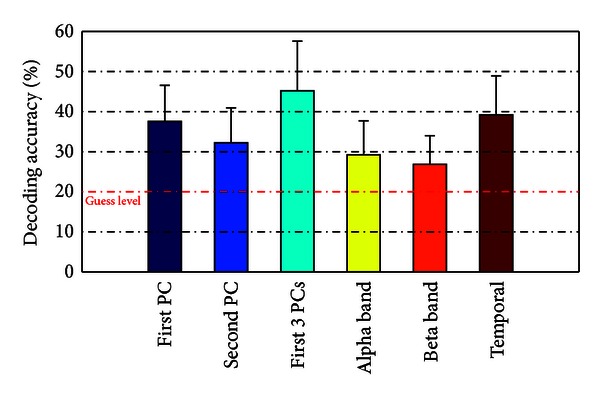
Comparison of decoding accuracies averaged over all subjects using different EEG features. The red dash dotted line indicates the guess level for 5-class classification problems.

Figure 4 summarizes the decoding accuracies in discriminating individual finger movements of one hand using differently computed features from EEG. Each bar in the figure presents the mean, together with the standard deviation, of decoding accuracies from all subjects and all permutations using one type of the features. The figure shows that all features produce average decoding accuracies above the guess level, which is indicated by the red dash dotted line. It also demonstrates that different features yield different average decoding accuracies, with projection coefficients on multiple spectral PCs, the highest (45.2%), followed by temporal data (39.2%), projection coefficients on single PCs (the first PC: 37.6% and the second PC: 32.2%), and then spectral powers in alpha band (29.3%) and beta band (26.8%). 

### 3.4. Evaluation of Decoding Performance

To evaluate decoding performance using different features, one-sample *t*-tests were firstly conducted between different features and the guess level. The results, as presented in Table 1, indicate most features produce decoding accuracies significantly higher than the guess level (*P* < 0.05), except for spectral powers in alpha band and in beta band. Comparison of decoding performance among different features was achieved by conducting pair-wise *t* tests on every two different features. The results in Table 1 demonstrate that projection coefficients on multiple spectral PCs produce an average decoding accuracy significantly higher than those achieved by all other features (*P* < 0.05). The decoding performance of temporal data is better than the single PC and spectral powers in both alpha and beta bands, while the difference is not significant as compared with data from the first PC (*P* > 0.05). Furthermore, the second PC (with combined features from both alpha and beta bands since it peaks on both frequency bands) indicates significantly better decoding accuracy than individual features from either alpha band or beta band.

**Figure 5:**
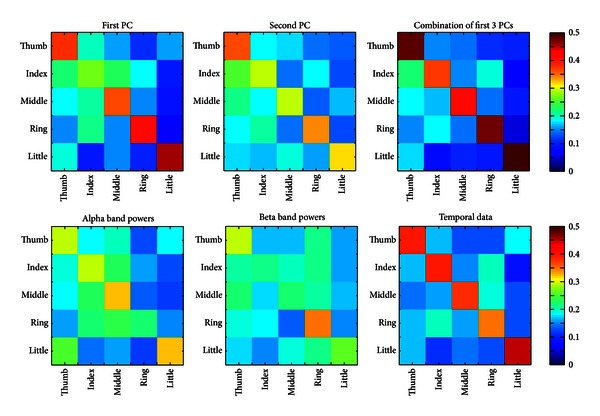
Confusion matrices for different types of EEG features. Horizontal axis: predicted labels for fingers; vertical axis: true labels for fingers.

To further examine the decoding performance of different features, confusion matrices for decoding each finger were computed (Figure 5). The present results indicate that entries on the main diagonal are most prominent for all features. It suggests that labels of trials in the testing set are mostly classified to the corresponding fingers correctly. It also can be observed that other large entries are mainly on either superdiagonal or subdiagonal lines of each confusion matrix, suggesting most misclassified trials were classified to neighbored fingers, rather than other fingers far away. When comparing confusion matrices from different features, the EEG feature of spectral PC project coefficients shows much less confusions than other EEG features, which is supported by decoding accuracy data. The EEG features from the first spectral PC projection coefficients and the temporal data have similar general performance in terms of confusion matrices, while these matrices further indicate different performance of these features on different fingers (e.g., the most confused finger is the index finger using first spectral PC projection coefficients and is the ring finger using temporal EEG data). The similar phenomenon is also observed in comparing other pairs of features. Again, the confusion matrix data indicate that the feature of alpha or beta band EEG signal changes has less power in discriminating different fingers. 

## 4. Discussion

In the development of noninvasive BCI applications, movement-related features are capable of providing BCI users with voluntary and intuitive control by extracting information from motor execution/imagination of certain body parts [45]. Movements of large body parts, such as hands, arms, and feet, have been successfully decoded using spectral powers from the low-frequency bands (i.e., alpha and beta bands) to generate control signals for noninvasive BCI [27–30, 46–48]. However, to develop BCI applications with rich complexity, the current available control features are not sufficient. Several BCI studies explored different aspects of movement-related information including projections from PCA decomposition and temporal waveforms [36, 37]. Their results suggest decoding movements from fine body parts, that is, individual finger movements, as one of promising approaches to improve the number of control features for BCI applications. These studies were carried out under different experimental conditions and using different signals, for example, invasive ECoG and noninvasive EEG, making it infeasible to compare the decoding performance of different features in discriminating individual finger movements from one hand in a unified configuration. The aim of present study is to evaluate features from a spectral PCA decomposition [37], spectral powers in individual frequency bands, and temporal data under the same protocol, that is, the discrimination of individual finger movements from one hand using noninvasive EEG.

For EEG features from spectral PCA decomposition, profiles and projections of the first three PCs are evaluated in the present study (Figure 2), since they account for most variations in data. The first PC shows a broadband phenomenon, which is consistent with the results in ECoG based BCI studies [37]. The result demonstrates, for the first time, that the features extracted from ECoG using spectral PCA decomposition also reside in EEG. In addition, the spatial patterns of projection coefficients on the first PC vary in movements of different fingers, suggesting that such a feature can be used to decode individual fingers from one hand. The resulting decoding accuracy that is significantly higher than the guess level (*P* < 0.05) further demonstrates the feasibility of using the broadband feature in EEG to discriminate individual fingers. Both the second and third PCs show large peaks in alpha and beta bands, which resemble ERD/ERS phenomena (particularly the second PC) [37] that are also demonstrated in EEG features of alpha/beta band spectral power changes (Figure 3(a)) in the present study. For EEG features of alpha/beta power changes, while they have demonstrated the promising performance in discriminating movements of large body parts in the literature [27–29], their decoding accuracies are not significantly higher than the guess level (*P* > 0.05) in discriminating individual fingers. This is consistent with the previous reports using ECoG data [37, 49] that low-frequency EEG components are more smeared and not spatially specific to individual fingers as compared with high-frequency EEG components (e.g., gamma band). It is worth noting that the broadband feature of the first spectral PC encompasses both low- and high-frequency EEG components. Lastly, the difference in EEG temporal data caused by movement of different fingers can be observed in averaged EEG data (Figure 3(b)). The present study further demonstrates the direct use of single-trial EEG temporal data (without any feature extraction techniques) in discriminating individual fingers, which shows comparable accuracies as reported in the literature [36].

These three types of EEG features have different discrimination performance in decoding individual fingers from one hand, as indicated by the data of decoding accuracy (Figure 4) and confusion matrix (Figure 5). In terms of decoding accuracy, the EEG feature from multiple spectral PCs yields the highest accuracy (i.e., 45.2% in decoding five fingers from one hand) among all studied features. The discrimination performance using multiple PCs exceeds those achieved from the use of any single PCs (*P* < 0.05), which suggest movement of fingers causes changes in EEG in a broad frequency range possibly contributed by multiple neural substrates, consistent with movement of other large body parts [27–29]. The better discrimination performance using multiple PCs further indicates movement-related changes in different frequency bands are independent, where all EEG frequency components contribute to the improved decoding accuracy. The discrimination performance using the first spectral PC is significantly better than the second PC (*P* < 0.05). The fact suggests that EEG spectral power changes in high-frequency bands (conveyed in the first PC) are more specific to individual finger movements than low-frequency EEG power changes [49, 50], similar to what is discussed above for ERD/ERS phenomena. Features from EEG temporal data produce the second best discrimination performance, which provides the direct evidence that useful information exists in noninvasive EEG data to decode individual fingers [36]. Its performance decrease as compared with the use of multiple spectral PCs might be caused by common changes presented in EEG data from all types of movements. It is noted that the use of the second PC yields significantly higher decoding accuracy than the guess level (*P* < 0.05) and higher decoding accuracy than individual alpha band or beta band power changes. Since the second PC resembles the combined phenomenon in both alpha and beta bands, the fact indicates the discrimination performance using EEG low-frequency components in decoding individual fingers can be improved when multiple frequency bands are integrated.

Meanwhile, it is important to note that the discrimination performance using EEG data is lower than those achieved by invasive measurements [33, 51, 52] due to relatively poor spatial resolution and low SNR of EEG. While the present study demonstrates a useful feature available in EEG to decoding individual fingers noninvasively, several practical factors can be considered to further improve the performance of EEG-based system in decoding fingers. EEG features can be enhanced by grouping and averaging a number of trials from the same fingers. Moreover, while a universe channel set is selected for different features to achieve a fair comparison in the present study, distinct channel sets should be investigated to acquire optimal discrimination performance. Furthermore, some spatial filtering algorithms, such as common spatial patterns (CSP) [53], are able to better reveal valuable spatial patterns of EEG features than simply selecting channel sets, which could improve the detection of features and, thus, the decoding performance.

## 5. Conclusion

The present study evaluated the discrimination performance from three types of EEG features, including spectral features obtained using PCA, alpha/beta band power changes, and EEG temporal data, in decoding individual finger movements from one hand. The experimental results demonstrate the feasibility of a broadband feature in EEG in discriminating individual fingers. Moreover, it is demonstrated that the use of multiple PCs (i.e., the first three PCs) can achieve the best decoding accuracy (45.2%) among all investigated EEG features. EEG temporal data yield a slightly lower decoding accuracy than spectral PC features. The present study further indicates that alpha/beta power changes do not contain sufficient information about fine individual finger movements, while they contribute to improved decoding accuracy when combined with other features. The findings in the present study provide a reference in selecting features for decoding individual fingers from one hand, which could largely increase the number of features for BCI applications and advance the state of the art of noninvasive BCI with rich complexity in control. 

## Figures and Tables

**Table 1:** Summary of *t*-test results on decoding accuracies using different features, as well as the guess level (20%). The bold entries indicate significant difference (*P* < 0.05).

	1st PC	2nd PC	First 3 PCs	Alpha band	Beta band	Temporal amplitudes	Guess level
1st PC	—	0.1390	**0.0470**	0.0595	**0.0191**	0.5030	**0.0092**
2nd PC	—	—	**0.0096**	0.5245	0.1552	**0.0290**	**0.0246**
First 3 PCs	—	—	—	**0.0310**	**0.0142**	**0.0446**	**0.0105**
Alpha band	—	—	—	—	0.2482	**0.0312**	0.0635
Beta band	—	—	—	—	—	**0.0089**	0.0763
Temporal amplitudes	—	—	—	—	—	—	**0.0099**
